# *Trapa japonica* Pericarp Extract Reduces LPS-Induced Inflammation in Macrophages and Acute Lung Injury in Mice

**DOI:** 10.3390/molecules21030392

**Published:** 2016-03-21

**Authors:** Yon-Suk Kim, Jin-Woo Hwang, Jae-Hyuk Jang, Sangkeun Son, Il-Bok Seo, Jae-Hyun Jeong, Ee-Hwa Kim, Sang-Ho Moon, Byong-Tae Jeon, Pyo-Jam Park

**Affiliations:** 1 Department of Biotechnology, Konkuk University, Chungju 27478, Korea; kimyonsuk@kku.ac.kr (Y.-S.K.); croucard@kku.ac.kr (J.-W.H.); 2 Chemical Biology Research Center, Korea Research Institute of Bioscience and Biotechnology, Ochang 28116, Korea; jangjh@kribb.re.kr (J.-H.J.); sonski@kribb.re.kr (S.S.); 3 Department of Anatomy, College of Oriental Medicine, Semyung University, Jecheon 27136, Korea; seoib@semyung.ac.kr; 4 Department of Food Science and Technology, Korea National University of Transportation, Jeungpyeong 27909, Korea; jhjeong@ut.ac.kr; 5 Department of Acupoint and Meridian, College of Oriental Medicine, Semyung University, Jecheon 27136, Korea; kimeh@semyung.ac.kr; 6 Nokyong Research Center, Konkuk University, Chungju 27478, Korea; moon0204@kku.ac.kr (S.-H.M.); hannokwon@kku.ac.kr (B.-T.J.)

**Keywords:** *Trapa japonica* pericarp, anti-inflammatory effect, bronchoalveolar lavage fluid (BALF), acute lung injury (ALI)

## Abstract

In this study, we found that chloroform fraction (CF) from TJP ethanolic extract inhibited lipopolysaccharide (LPS)-induced production of nitric oxide (NO) and intracellular ROS in RAW264.7 cells. In addition, expression of cyclooxygenase-2 (COX-2) and inducible nitric oxide synthase (iNOS) genes was reduced, as evidenced by western blot. Our results indicate that CF exerts anti-inflammatory effects by down-regulating expression of iNOS and COX-2 genes through inhibition of MAPK (ERK, JNK and p38) and NF-κB signaling. Similarly we also evaluated the effects of CF on LPS-induced acute lung injury. Male Balb/c mice were pretreated with dexamethasone or CF 1 h before intranasal instillation of LPS. Eight hours after LPS administration, the inflammatory cells in the bronchoalveolar lavage fluid (BALF) were determined. The results indicated that CF inhibited LPS-induced TNF-α and IL-6 production in a dose dependent manner. It was also observed that CF attenuated LPS-induced lung histopathologic changes. In conclusion, these data demonstrate that the protective effect of CF on LPS-induced acute lung injury (ALI) in mice might relate to the suppression of excessive inflammatory responses in lung tissue. Thus, it can be suggested that CF might be a potential therapeutic agent for ALI.

## 1. Introduction

Inflammation is a complex biological response to protect against a wide range of injuries caused by infectious agents, antigen challenge, and physical, chemical or traumatic damage [1]. De-regulation and perpetual activation of inflammation can lead to many diseases, such as rheumatoid arthritis, chronic asthma, multiple sclerosis, inflammatory bowel disease and psoriasis [2]. One of the most potent stimuli to macrophages is the bacterial endotoxin lipopolysaccharide (LPS), which induces the production of a variety of cytokines and inflammatory mediators including nitric oxide (NO), prostaglandin E2 (PGE2), interleukin 1β (IL-1β) and tumor necrosis factor-α (TNF-α) [3,4]. *Trapa japonica* has been used as ethno-medicine for the treatment of gastric ulcer, diarrhea, hangover after alcohol consumption, and dysentery. Various other pharmacological activities of *Trapa japonica* include, antioxidant activity, hepatoprotective activity, and antidiabetic activity [5,6]. The pericarp of *Trapa japonica* contains many dietary fibers [7] and phenolic compounds such as 1,2,3,6-*tetra*-*O*-galloyl-beta-d-glucopyranose (TGG), trapain, ellagic acid, eugeniin and gallic acid [6,8]. Although *Trapa japonica* has long been used in folk medicine, little has been explored about the physiological functions of its active ingredients.

In the present study, we determined the effects of the chloroform fraction (CF) from *Trapa japonica* pericarp in LPS-induced inflammatory response in RAW264.7 cells, particularly, by estimating NO, ROS and cytokine production, and exploring their related signaling pathways. Acute lung injury (ALI) and its severe form, acute respiratory distress syndrome (ARDS), are clinical syndromes characterized by lung inflammation and increased microvascular permeability [9]. A variety of clinical disorders such as pneumonia, aspiration of gastric contents, sepsis, major trauma and acute pancreatitis can lead to induction of ALI [10]. Many experimental animal models causing ALI have been established to investigate the precise mechanisms behind this disorder [11,12]. Among them, intranasal instillation of LPS is widely used to induce lung injury because it yields very reproducible results [13]. LPS directly stimulates neutrophils to migrate into the lungs, with additional recruitment by LPS-induced pro-inflammatory mediators such as TNF-α [14]. These pro-inflammatory mediators play an important role in the development of ALI [15]. Hence these, inflammatory mediators may serve as therapeutic targets for treating lung injury. In the present study, experimental ALI was induced by LPS, and the effects of the chloroform fraction from *Trapa japonica* pericarp (CF) on inflammation were determined both *in vitro* and *in vivo*.

## 2. Results

### 2.1. Active Compounds

All the active compounds from the CF were identified. The six active compounds identified as gallic acid (MW: 170.1), 1,2,3-trigalloyl glucoside (MW: 636.0), 1,6-digalloyl glucoside (MW: 484.0), 1,2,6-trigalloyl glucoside (MW: 636.0), eugeniin (MW: 938.6) and 1,2,3,6,-tetragalloyl glucoside (MW: 788.5) were also reported as active principles by Shindo *et al.* [8]. Additionally we have discovered and isolated for the first time two more compounds from CF, namely 3.9-dihydroxydibenzo-[*b*,*d*]pyran-6-one and 3-hydroxy-6*H*-dibenzo[*b*,*d*]pyran-6-one (also known as 3-hydroxy-6*H*-benzo-[*c*]chromen-6-one, 3-hydroxy-6*H*-dibenzo[*b*,*d*]pyran-6-one, 3-hydroxydibenzo-α-pyrone or 3-hydroxyurolithin. These eight active ingredients have been documented to have anti-oxidant activities [8] and it can be speculated that these compounds might have anti-inflammatory activities as well.

### 2.2. NO Assay

**Figure 1:**
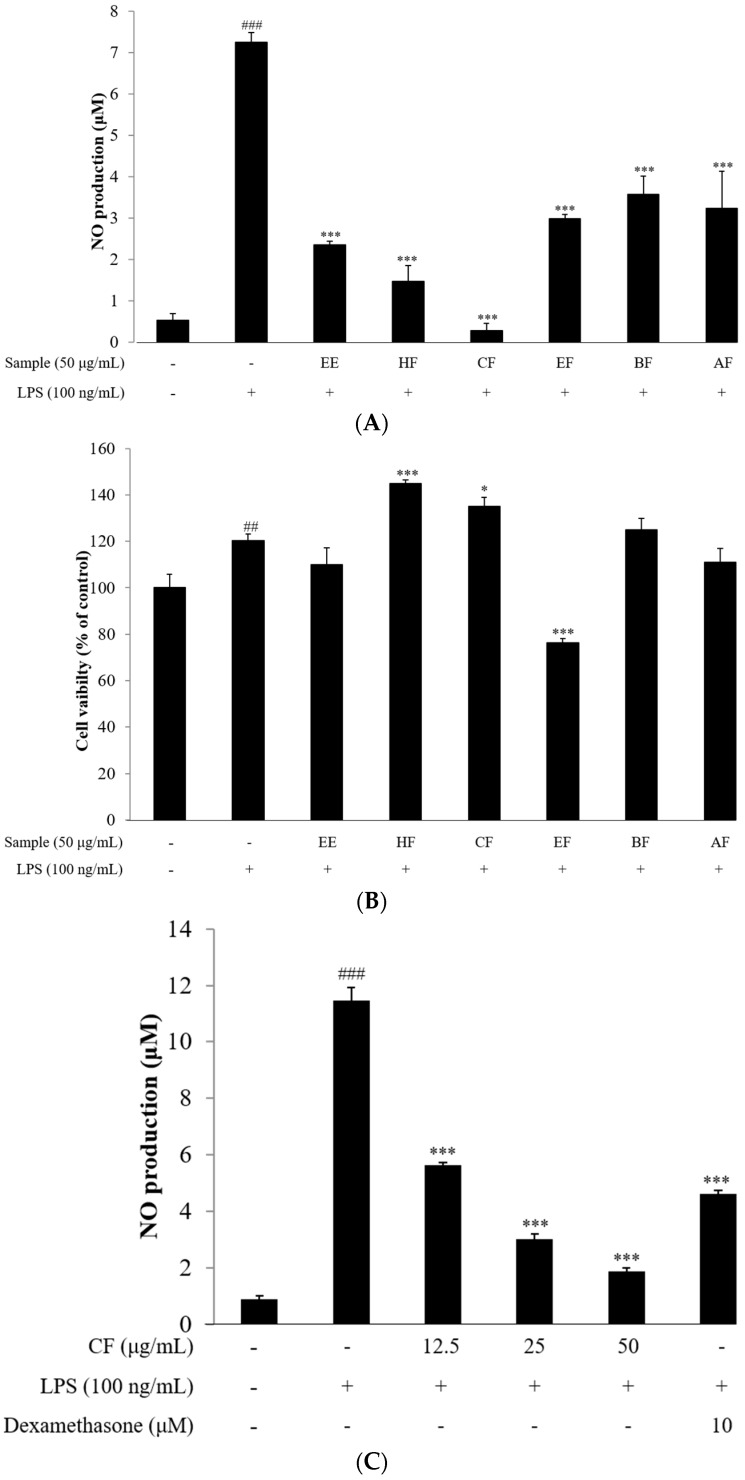

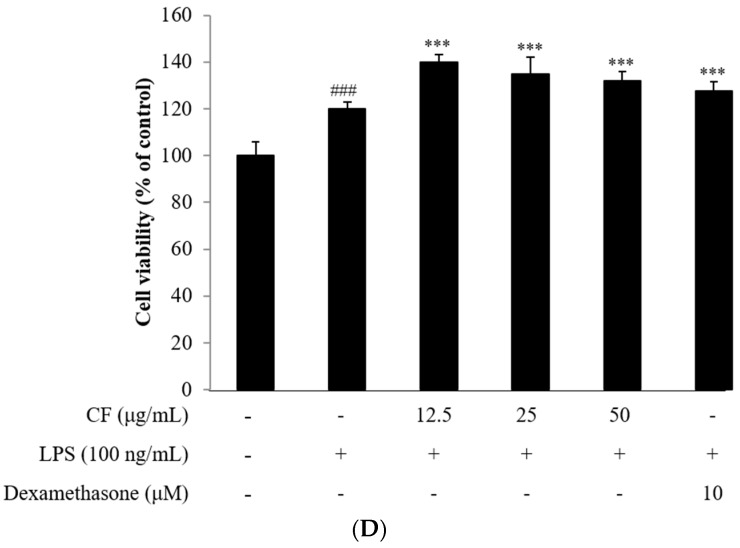
Effect of *Trapa japonica* pericarp and CF on NO release and cell toxicity. (**A**,**C**) Inhibitory effects of solvent-partitioned layers of *Trapa japonica* pericarp and CF on NO production in RAW264.7 cells. The ethanol extract of *Trapa japonica* pericarp was partitioned into hexane, chloroform, ethyl acetate, *n*-butanol, aqueous fractions (50 μg/mL) and CF (12.5, 25 and 50 μg/mL), then tested for inhibitory effects on LPS-induced production of NO using the Griess reagent; (**B**,**D**) Cell viability of RAW264.7 cells when treated with solvent-partitioned layers of *Trapa japonica* pericarp and CF. The ethanol extract of *Trapa japonica* pericarp was partitioned into hexane, chloroform, ethyl acetate, *n*-butanol, aqueous fractions (50 μg/mL), CF (12.5, 25 and 50 μg/mL) and then tested for cytotoxicity using the MTT assay and dexamethasone (10 μM) as reference. Cells were pretreated with the samples for 1 h, then exposed to LPS treatment for 20 h. Data are expressed as the means ± SD of three different samples. ^##^ *p* < 0.01, ^###^ *p* < 0.01 *vs.* control group, * *p* < 0.05 and *** *p* < 0.001 *vs.* LPS group.

Firstly, we investigated the effect of the ethanolic extracts of *Trapa japonica* pericarp (EE), and its fractions (hexane (HF), chloroform (CF), ethyl acetate (EF), *n*-butanol (BF) and aqueous (AF)) on LPS-induced production of NO in RAW264.7 cells. The level of NO significantly increased after RAW264.7 cells were provided LPS-stimulation for 18 h. At 50 μg/mL, all samples of ethanolic extracts of *Trapa japonica* pericarp (EE), and its fractions demonstrated significant reduction the NO production (Figure 1A).

### 2.3. Cell Viability

The potential cytotoxicity of EE, HF, CF, EF, BF and AF at 50 μg/mL was evaluated using the MTT assay. The results elicited that, apart from EF, the cell viability was not affected by any of the fractions at 50 μg/mL (Figure 1B). Furthermore, the cytotoxicity of various concentrations of CF was examined, and CF was observed not to affect the cell viability of LPS-stimulated RAW264.7 cells (Figure 1D). Among the different fractions, the CF fraction was selected for testing in further experiments since it displayed the highest inhibition of NO in a dose-dependent manner (Figure 1C) without any significant cytotoxicity to the RAW264.7 cells.

### 2.4. Cytokine Assay

**Figure 2:**
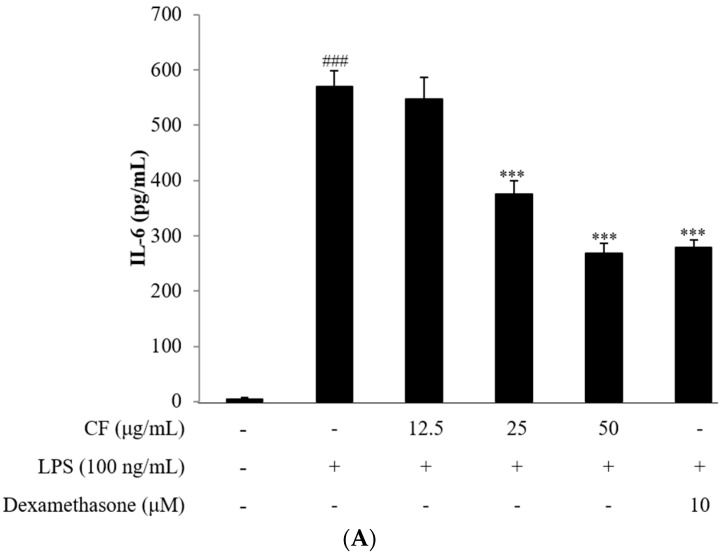

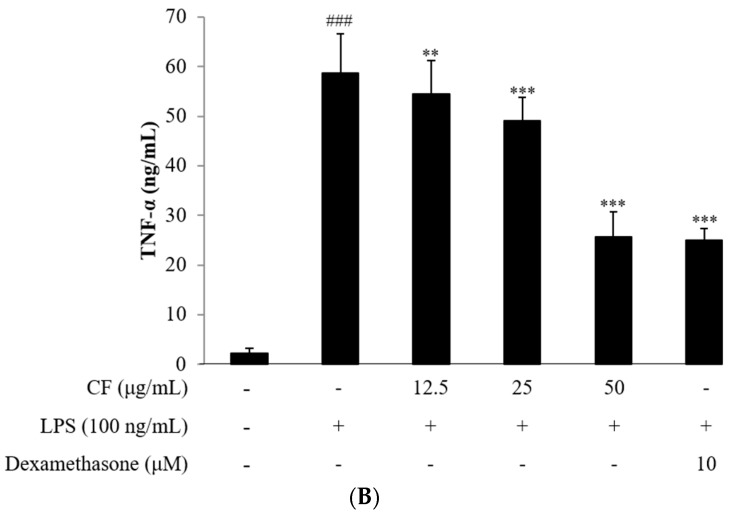
Inhibitory effects of the CF on LPS-induced production of pro-inflammatory cytokines (**A**) IL-6; (**B**) TNF-α. Release of the pro-inflammatory cytokine into the supernatants from RAW264.7 cells incubated with the CF (12.5, 25 and 50 μg/mL) and dexamethasone (10 μM) with or without LPS (100 ng/mL) for 18 h were examined by the ELISA method. Data are expressed as the means ± SD of three different samples. ^###^ *p* < 0.01 *vs.* control group, ** *p* < 0.01, *** *p* < 0.001 *vs.* LPS group.

Overproduction of IL-6 and TNF-α by RAW264.7 macrophages was generally observed after stimulation with LPS for 18 h. However, pretreatment with CF inhibited the production of both TNF-α and IL-6 in a dose dependent manner (Figure 2A,B).

### 2.5. Measurement of Intracellular ROS

**Figure 3:**
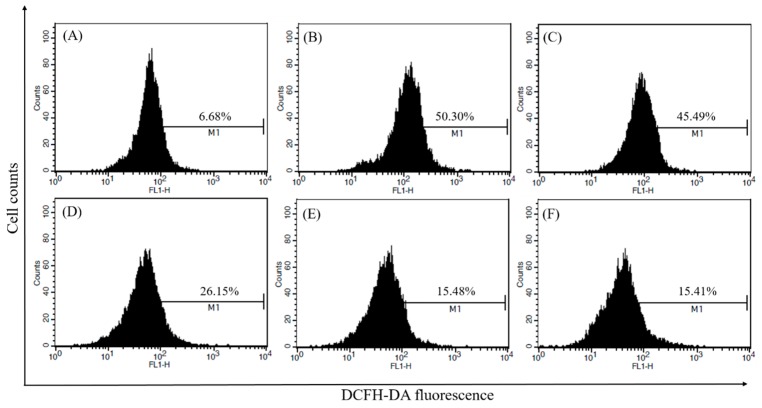
Measurement of intracellular ROS. RAW264.7 cells were pretreated with CF for 1 h and incubated with LPS (100 ng/mL) for 30 min. The medium was then replaced with fresh medium containing DCFH-DA (10 μM). After 30 min, the level of intracellular ROS was monitored using flow cytometry. (**A**) Control; (**B**) LPS (100 ng/mL); (**C**) LPS (100 ng/mL) + CF (12.5 μg/mL); (**D**) LPS (100 ng/mL) + CF (25 μg/mL); (**E**) LPS (100 ng/mL) + CF (50 μg/mL); and (**F**) LPS (100 ng/mL) + NAC (20 mM). Values are expressed as the mean ± SD of three independent experiments.

LPS-induced intracellular ROS generation was determined in RAW264.7 cells using the ROS sensitive fluorescent dye DCFH-DA. CF was tested for the ability to inhibit the intracellular ROS generation in RAW264.7 cells stimulated with LPS. The percentage of DCFH-DA treated fluorescent cells was 50.30% after LPS treatment, but pretreatment with different concentration of CF decreased the fluorescence to 45.49% (12.5 μg/mL), 26.15% (25 μg/mL), and 15.48% (50 μg/mL), respectively (Figure 3). As a positive control, NAC (20 mM) demonstrated a significant decrease in the intracellular ROS (15.41%). Altogether, pretreatment with CF strongly inhibited the LPS-mediated increase in intracellular ROS level in a dose dependent manner.

### 2.6. iNOS and COX-2 Expression

**Figure 4:**
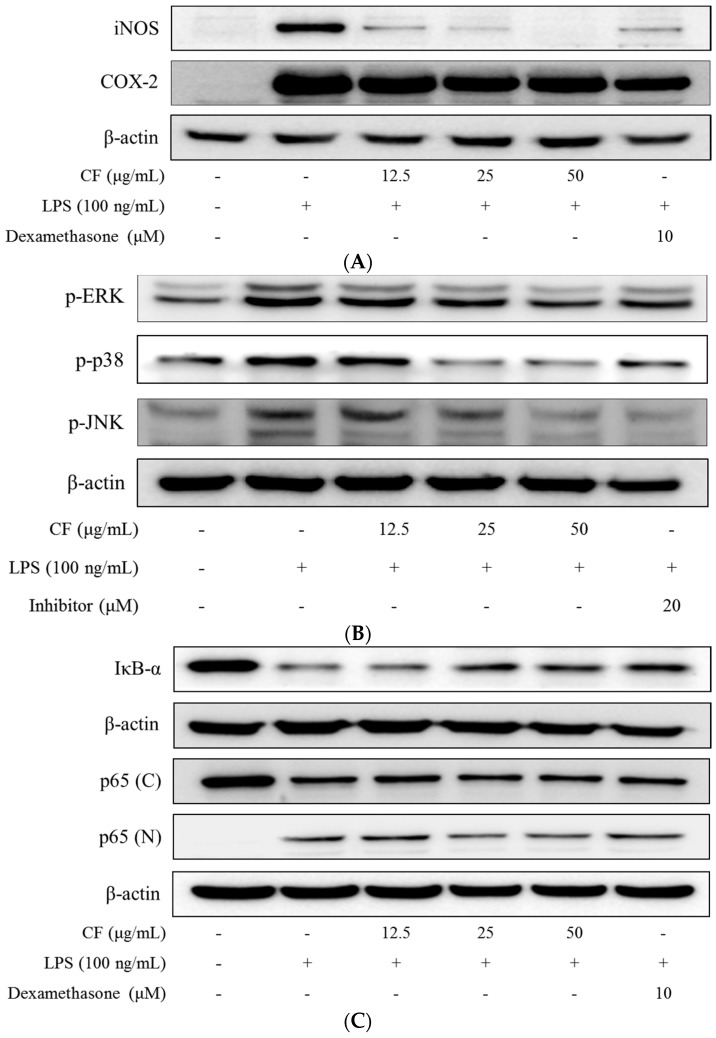

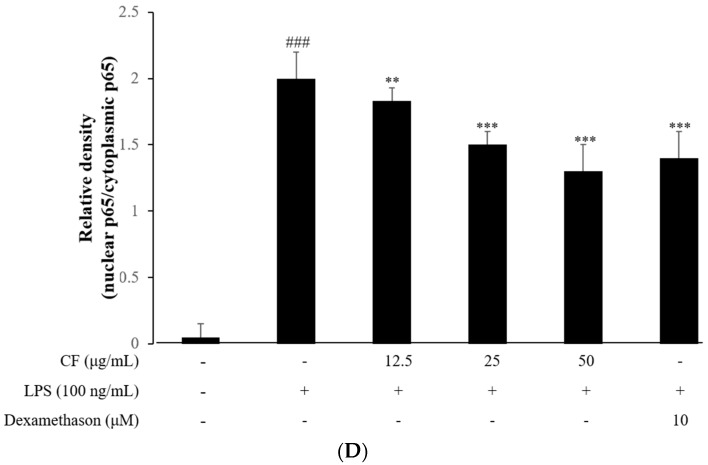
(**A**) Inhibitory effects of the CF on LPS-induced iNOS and COX-2 expression. Expression of iNOS and COX-2 protein in RAW264.7 cells incubated with the CF (12.5, 25 and 50 μg/mL) and dexamethasone (10 μM) in the presence or absence of LPS (100 ng/mL) for 18 h were examined by western blot analysis; (**B**) Inhibitory effects of the CF on LPS-induced activation of MAPKs. Activation of MAPKs, including ERK, p38 and JNK, in RAW264.7 cells incubated with the CF (12.5, 25 and 50 μg/mL) and inhibitor in the presence or absence of LPS (100 ng/mL) for 30 min was examined by western blot analysis. Cells were treated with 20 μM of the MAPK inhibitors PD098059 (ERK inhibitor), SB203580 (p38 inhibitor), and SP600125 (JNK inhibitor); (**C**) Effects of CF on the LPS-induced phosphorylation of IκB-α and NF-κB activation; (**D**) Relative density of nuclear p65/cytoplasmic p65. RAW264.7 macrophages were pretreated with CF (12.5, 25 and 50 μg/mL) and dexamethasone (10 μM) for 1 h, and then stimulated with LPS (100 ng/mL) for 15 min. Total cellular proteins were prepared and western blotted for IκB-α. Nuclear (N) and cytosolic (C) extracts were isolated, and the levels of p65 were determined by western blotting.

Expression of iNOS protein was minute in unstimulated RAW264.7 cells, but considerable up-regulation was observed upon exposure to LPS for 18 h. CF at the concentrations of 12.5, 25 and 50 μg/mL caused a dose-dependent decrease in the expressions of both iNOS and COX-2 protein in RAW264.7 cells after LPS stimulation for 18 h (Figure 4A).

### 2.7. Inhibition of MAPKs Phosphorylation

To elucidate the effects of CF on the MAPK pathway, the phosphorylation of ERK, JNK, and p38 was examined. Cell lysates of RAW264.7 cells were analyzed by western blot with anti-phosphorylated ERK, anti-phosphorylated JNK, or anti-phosphorylated p38 antibody. LPS treatment markedly activated MAPKs, including JNK, p38 and ERK (Figure 4B), however, pretreatment with CF significantly reduced the LPS-induced phosphorylation of JNK, p38 and ERK in a dose-dependent manner.

### 2.8. IκB-α Degradation

IκB-α degradation is the key step in NF-κB activation, hence the effects of CF on IκB-α degradation was next investigated. Compared with the unstimulated RAW264.7 cells, IκB-α quickly decreased after 15 min of LPS stimulation, while the pre-treatment with CF at concentrations of 12.5, 25 and 50 μg/mL allowed significant recovery of the LPS-induced degradation of IκB-α (Figure 4C). NF-κB activation is a critical event in the activation of several inflammatory genes, including iNOS and COX-2 [16]. These results indicated that the molecular mechanism for the CF-mediated attenuation of LPS-induced inflammatory responses in RAW264.7 cells could be related to the inhibition of IκB-α phosphorylation and degradation, and further nuclear, the translocation of p65, which in turn leads to the suppression of inflammatory mediators (iNOS, COX-2, TNF-α and IL-6).

### 2.9. Histological Study

**Figure 5:**
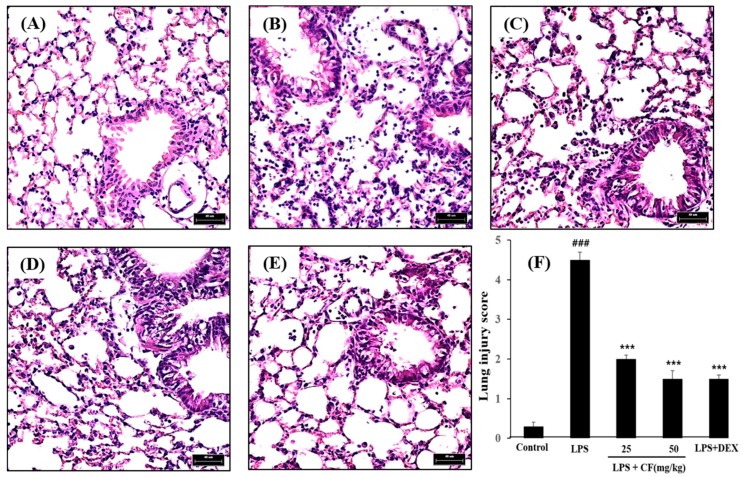
Effects of CF on histopathologic changes in lung tissues in LPS-induced ALI mice using hematoxylin-eosin (HE) staining. LPS induced inflammatory cell infiltration in the lung parenchyma compared to control mice, while CF attenuated the infiltration of inflammatory cells in the lung compared to the LPS-treated mice. The images are representative of lung specimens for each condition: (**A**) Control group; (**B**) LPS (5 mg/kg) group; (**C**) LPS + CF (25 mg/kg) group; (**D**) LPS + CF (50 mg/kg) group; and (**E**) LPS + dexamethasone (10 mg/kg) group; Scale bar, 40 μm; (**F**) Lung injury scores were calculated according to the sum of the levels of cell infiltration and damage levels as assessed from the lung sections. The values presented are the mean ± SD of three independent experiments (*n* = 4 in each group). ^###^ *p* < 0.01 *vs.* control group, *** *p* < 0.001 *vs.* LPS group.

As shown in Figure 5, characteristic morphological changes were observed in lung sections after LPS administration, including infiltration of inflammatory cells, capillary congestion, hemorrhage, and marked thickening of the alveolar wall. The morphological changes in the lung tissue of the mice with ALI suggested that the ALI model was successfully triggered under the present experimental conditions. The CF group, at doses of 25 and 50 mg/kg, and the dexamethasone group, at the dose of 10 mg/kg, showed significant reduction in the LPS-induced lung tissue damage. These results suggested that CF possessed a protective effect for the LPS-induced ALI.

### 2.10. Total Protein Content Determination in BALF

**Figure 6:**
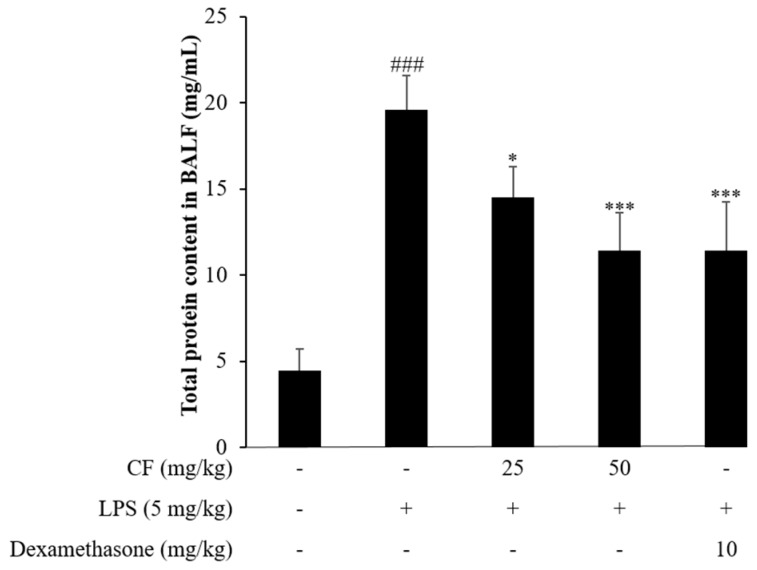
Effects of CF on the total protein content in the BALF of LPS-induced ALI mice. Mice were given an intraperitoneal injection of CF (25 and 50 mg/kg) or dexamethasone (10 mg/kg) twice, 24 h and 2 h before the intranasal administration of LPS. BALF was collected 24 h after the LPS challenge for analysis of the total protein content using Bradford assay. The values presented are the means ± SD. ^###^ *p* < 0.01 *vs.* control group, * *p* < 0.05, *** *p* < 0.001 *vs.* LPS group.

The inhalation of LPS group caused a significant 4.4-fold increase in the total protein content (Figure 6) when compared to control group. Injection of CF group significantly reduced LPS-induced elevation in total protein content in BALF. Our result showed that LPS caused a marked increase in total protein content in BALF, this observation is in agreement with the previous study [17] and CF may prevent LPS-induced ALI mice model.

### 2.11. Cell Counts in BALF

**Figure 7:**
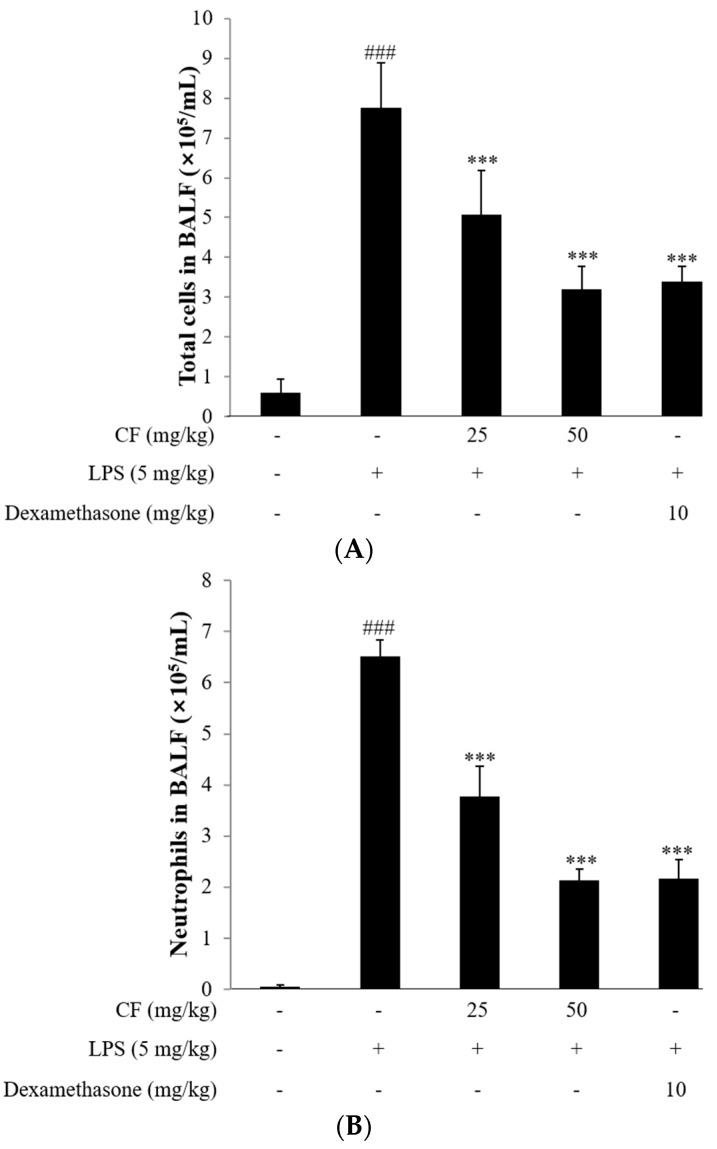

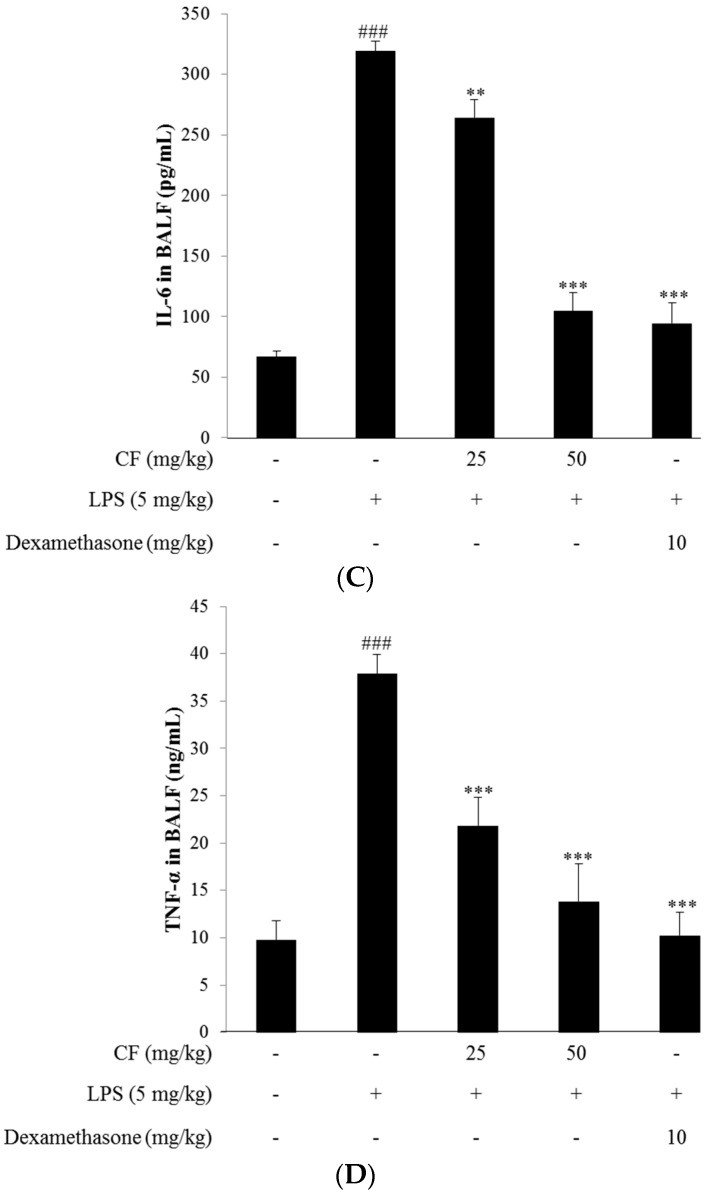
Effects of CF on (**A**) total cell; (**B**) neutrophils counts, inflammatory cytokine (**C**) IL-6 and (**D**) TNF-α production in the BALF of LPS-induced ALI mice. Mice (*n* = 8) were given an intraperitoneal injection of CF (25 and 50 mg/kg) or dexamethasone (10 mg/kg) twice, 24 h and 2 h before intranasal LPS administration. BALF was collected 24 h after the LPS challenge for analysis. The values presented are the means ± SD. ^###^ *p* < 0.01 *vs.* control group, ** *p* < 0.01, *** *p* < 0.001 *vs.* LPS group.

The number of inflammatory cells, such as neutrophils, present in the BALF was detected. After LPS challenge, the accumulation of total cells and neutrophils significantly increased compared to the control group (*p* < 0.001); however, the groups pretreated with CF (25 and 50 mg/kg, *p* < 0.001) and dexamethasone (10 mg/kg, *p* < 0.001) displayed a significant decrease in the level of total cells and neutrophils in the BALF. As shown in Figure 7A,B, LPS challenge significantly increased the number of total cells (7.7 ± 1.2 × 10^5^/mL) and neutrophils (6.5 ± 0.3 × 10^5^/mL) compared with the control group (*p* < 0.001). Meanwhile, the CF group was found to have a significant decrease in the number of total cells (25 mg/kg, 5.0 ± 1.1 × 10^5^/mL, 50 mg/kg, 3.2 ± 0.5 × 10^5^/mL) and neutrophils (25 mg/kg, 3.7 ± 0.5 × 10^5^/mL, 50 mg/kg, 2.1 ± 0.4 × 10^5^/mL), which occurred in a dose-dependent manner. Especially, the 50 mg/kg treatment of the CF group showed similar data in comparison with the dexamethasone group, used as a positive control.

### 2.12. Cytokine Measurement in BALF

In this study, the levels of the cytokines TNF-α and IL-6 in the BALF were examined to further reveal the anti-inflammatory effects of CF on the mice with ALI. As shown in Figure 7C,D, the concentrations of the pro-inflammatory cytokines TNF-α and IL-6 significantly increased in the BALF of mice exposed to LPS when compared to the control group (*p* < 0.001); however, both doses of the CF group (25 mg/kg, *p* < 0.01 and 50 mg/kg, *p* < 0.001) and the dexamethasone group (10 mg/kg, *p* < 0.001) displayed marked suppression of IL-6 expression.

## 3. Discussion

LPS-stimulation of macrophages disrupts the balance of the intracellular reduction-oxidation state, which leads to oxidative stress [18]. NO can mediate the functions of many types of cells at the site of inflammation, including T lymphocytes, macrophages, synovial fibroblasts, osteoclasts, and endothelial cells [19]. Meanwhile, excessive NO may promote cytokine and matrix metalloproteinase production, mitochondrial dysfunctions and cell apoptosis which accelerate the development of inflammation [20]. Therefore, NO has been considered as a promising therapeutic target for the treatment of inflammatory diseases.

In the present study, we investigated the remarkable inhibitory effects of the chloroform fraction from *Trapa japonica* pericarp (CF) on NO production by LPS-stimulated RAW264.7 cells. We demonstrated that CF decreased the expression of iNOS protein in a dose-dependent manner. It was previously reported that the inhibition of NO production was mainly due to the inhibition of iNOS protein expression [21]. Several intracellular signaling pathways are involved in the regulation of the inflammatory reaction in LPS-stimulated macrophages, such as the MAPKs pathway, which is linked to the activation of the transcription factor NF-κB [22]. MAP kinases are a group of serine/threonine protein kinases comprising three subfamilies: the ERKs, JNKs, and the p38 MAPKs [23]. MAPKs can be activated by various extracellular molecules, and can induce the phosphorylation of many key signaling molecules related to cell proliferation, inflammation, and apoptosis [23]. Our results displayed that CF attenuated the phosphorylation of p38, ERK, and JNK, reducing the subsequent inflammatory response. The association of the NF-κB p65/p50 dimer with IκB-α plays a pivotal role in regulating its nuclear translocation and gene transcription [24]. After exposure of RAW264.7 cells with LPS, IκB-α degradation caused the dissociation and nuclear translocation of p65. We analyzed the effects of CF on IκB-α in LPS-stimulated RAW264.7 cells, and the results exhibited that CF suppressed the LPS-induced degradation of IκB-α and consequently, the nuclear translocation of p65. Taken together, our findings suggest that CF might reduce the LPS-induced production of NO by RAW264.7 macrophages through the inhibition of iNOS protein expression. CF was also found to inhibit the production of pro-inflammatory cytokines (IL-6 and TNF-α), and to suppress the LPS-induced phosphorylation of MAPKs, and NF-κB activation, particularly upon IκB-α degradation.

Excessive accumulation and activation of neutrophils in the alveolar space is the characteristic of pulmonary inflammation [25]. In this study, we found that pretreatment with CF suppressed the LPS-induced over-production of neutrophils in the BALF. Aside from neutrophils, TNF-α, IL-1β and IL-6, the principal pro-inflammatory cytokines, are also involved in the pathophysiology of endotoxin-induced ALI [26]. These cytokines are crucial mediators in a range of acute and chronic responses to inflammatory diseases [27]. CF also demonstrated inhibition of cytokine production. The results of this study, therefore suggested that treatment with CF might improve the lung function by inhibiting inflammation through the down regulation of TNF-α and IL-6. These anti-inflammatory effect of CF from *Trapa japonica* pericarp may be attributed to the following active compounds: gallic acid, 1,2,3-trigalloyl glucoside, 1,6-digalloyl glucoside, 1,2,6-trigalloyl glucoside, eugeniin, 1,2,3,6,-tetragalloyl glucoside, 3,9-dihydroxydibenzo[*b*,*d*]pyran-6-one and 3-hydroxy-6*H*-dibenzo-[*b*,*d*]pyran-6-one.

## 4. Materials and Methods

### 4.1. Materials

DMEM medium and fetal bovine serum (FBS) were purchased from Life Technologies, Inc. (Carlsbad, CA, USA). Lipopolysaccharide (LPS) isolated from *Escherichia coli* 0111:B4 and dexamethasone were purchased from Sigma-Aldrich (St. Louis, MO, USA). TNF-α and IL-6 ELISA kits were purchased from R & D Systems (Minneapolis, MN, USA). The antibodies against inducible NO synthase (iNOS), COX-2, IκB-α, phospho-p38, phospho-JNK, phospho-ERK, and β-actin were purchased from Cell Signaling Technology (Beverly, MA, USA). Ly-6G (Gr-1) was purchased from eBioscience (San Diego, CA, USA). All other reagents were of the highest grade commercially available.

### 4.2. Extraction and Isolation

The *Trapa japonica* pericarp was obtained at the Jecheon herbal medicine mall (Jecheon, Korea). Its pericarp was obtained using a straw cutter and a milling treatment. The *T. japonica* pericarp (6 kg) were extracted with 70% EtOH (2 × 50 L) under reflux. After removal of the solvent under reduced pressure, the residue (30 g) was suspended in H_2_O (600 mL) and successively partitioned with hexane (3 × 600 mL), CHCl_3_ (3 × 600 mL), EtOAc (3 × 600 mL), and *n*-BuOH (3 × 600 mL). Active compounds were then isolated and purified from the crude *Trapa japonica* pericarp extracts by semi-preparative high-performance liquid chromatography (HPLC). HPLC was performed using an Ultimate 3000 system (Thermo Scientific, Waltham, MA, USA) equipped with a UV detector. The column and mobile phase consisted of a Semi-Prep column (250 × 20 mm) fitted with a guard module. The mobile phase was composed of 0.1% acetic acid in water (A) and 100% MeOH (B). The gradient conditions were as follows: for 0–5 min, the content of mobile phase B was increased linearly from 0% to 60% and for 5–50 min the content of mobile phase B was increased linearly from 60% to 100%. The flow rate was 5 mL/min, injection volume was 1 mL and wavelength was 254 nm. The fractions were separated according to their graphic peak into three main fractions. All solvents and samples were filtered through a 0.22 μm Millipore filter. Fractions CHCl_3_-II, III were separated by reversed-phase HPLC (Cosmosil C18, 10 × 250 mm, 10 μm) using a linear gradient solvent system of 40%–65% CH_3_CN/H_2_O over 25 min at a flow rate of 3 mL/min, resulting in the isolation of two compounds (*t*_R_ = 12.6 min). 3,9-Dihydroxydibenzo[*b*,*d*]pyran-6-one, the molecular formula: C_13_H_8_O_4_, positive ESI-MS *m*/*z*: 229.1 [M + H]^+^; ^1^H-NMR (700 MHz, CD_3_OD) δ 8.02 (d, *J* = 8.8 Hz, 1H, H-1), 6.81 (dd, *J* = 8.8, 2.3 Hz, 1H, H-2), 7.23 (d, *J* = 2.2 Hz, 1H, H-4), 7.89 (d, *J* = 8.8 Hz, 1H, H-7), 6.80 (dd, *J* = 8.7, 2.4 Hz, 1H, H-8), 6.67 (d, *J* = 2.4 Hz, 1H, H-10); ^13^C-NMR (176 MHz, CD_3_OD) δ 132.06 (C-1), 118.50 (C-2), 170.1 (C-3), 106.73 (C-4), 138.11 (C-5), 110.19 (C-6), 123.84 (C-7), 112.84 (C-8), 160.28 (C-9), 102.71 (C-10). 152.69 (C-11), 163.00 (C-13), 107.6 (C-14). 3-Hydroxy-6*H*-dibenzo[*b*,*d*]pyran-6-one, the molecular formula: C_13_H_8_O_3_, positive ESI-MS *m*/*z*: 213.3 [M + H]^+^; ^1^H-NMR (800 MHz, CD_3_OD) δ 8.26 (dd, *J* = 7.9, 1.0 Hz, 1H, H-8), 8.16 (d, *J* = 8.1 Hz, 1H, H-4), 8.05 (d, *J* = 8.7 Hz, 1H, H-7), 7.84 (ddd, *J* = 8.3, 7.2, 1.4 Hz, 1H, H-3), 7.52 (td, *J* = 7.7, 1.0 Hz, 1H, H-2), 6.86 (dd, *J* = 8.7, 2.4 Hz, 1H, H-8), 6.74 (d, *J* = 2.4 Hz, 1H, H-10); ^13^C-NMR (200 MHz, CD_3_OD) δ 163.54 (C-1), 161.63 (C-9), 154.04 (C-11), 137.20 (C-5), 136.51 (C-3), 131.22 (C-1), 128.72 (C-2), 125.66 (C-7), 122.60 (C-4), 120.74 (C-14), 114.55 (C-8), 111.43 (C-6), 104.34 (C-10).

### 4.3. In Vitro Study

#### 4.3.1. Cell Culture

RAW264.7 macrophages were cultured in Dulbecco’s modified Eagle’s medium (DMEM) supplemented with heat-inactivated 10% fatal bovine serum (FBS), 100 U/mL of penicillin and 100 μg/mL of streptomycin at 37 °C in a humidified atmosphere with 5% CO_2_. In all experiments, macrophages were incubated in the presence or absence of various concentrations of the chloroform fraction (CF) from *Trapa japonica* pericarp which were always added 1 h prior to LPS (100 ng/mL) stimulation. The CF was dissolved in ethanol and was subjected to serial dilution so that the final concentration of ethanol in solution was less than 0.5%.

#### 4.3.2. Cell Viability

Cell viability was determined by an MTT assay. RAW264.7 macrophages were pretreated with different concentrations of CF (12.5, 25 and 50 μg/mL) for 1 h, then exposed to LPS (100 ng/mL) treatment for 20 h. After 20 h of incubation, 0.5 mg/mL MTT was added to each well, and the cells were incubated for another 2 h at 37 °C with 5% CO_2_. The supernatants were then aspirated, and the formazan crystals in each well were dissolved in 150 μL of DMSO. Absorbance was measured by microplate reader at a wavelength of 540 nm. The optical density of MTT-formazan formed in untreated cells was taken as 100% viability.

#### 4.3.3. Measurement of NO Production

Nitric oxide (NO) in the RAW264.7 cell culture supernatant was determined via reaction with Griess reagent. The supernatant was collected, and mixed with equal amounts of Griess reagent (containing 0.1% *N*-(1-naphthyl)ethylenediamine, 1% sulfanilamide and 5% phosphoric acid), then shaken lightly for 10 min at room temperature. Finally, the absorbance value of triplicate samples was read with a microplate reader using a test wavelength of 540 nm and nitrite concentration was determined using a dilution of sodium nitrite as the standard.

#### 4.3.4. Measurement of Pro-Inflammatory Cytokine Production

RAW264.7 cells were plated in 24-well plates (4 × 10^5^ cells/well) and incubated in the presence of either LPS alone, or LPS + CF (12.5 μg/mL, 25 μg/mL and 50 μg/mL) for 18 h. Dexamethasone (10 μM) was used as a positive control. Cell-free supernatants were subsequently used in the pro-inflammatory cytokine assays, which were carried out with a mouse enzyme-linked immunosorbent assay (ELISA) kit (R D Systems), according to the manufacturer’s instructions.

#### 4.3.5. Measurement of Intracellular ROS

The LPS-induced intracellular ROS generation by RAW264.7 cells was determined using the ROS sensitive fluorescent dye DCFH-DA. To observe intracellular ROS production through the oxidation of DCFH-DA, near confluent cells in 6-well plates were pretreated with CF (12.5 μg/mL, 25 μg/mL and 50 μg/mL) for 1 h and then incubated with LPS (100 ng/mL) for 18 h, followed by with DCFH-DA (1 mM) for 30 min. Cells were washed twice with PBS, and the intracellular levels of ROS were analyzed by flow cytometry.

#### 4.3.6. Western Blotting

After treatment with CF and LPS, the cells were washed with ice-cold phosphate-buffered saline (PBS) and then harvested, and lysed immediately. Cytoplasmic and nuclear extracts were prepared with Nuclear and Cytoplasmic kits (Thermo, Rockford, CA, USA) based on the manufacturer’s instructions. The total protein concentration was determined with a Bradford protein assay kit. After appropriate treatment, the cells were washed with PBS and lysed in cold lysis buffer. Following lysis, cells were mixed with SDS/PAGE loading buffer and boiled for 5 min at 100 °C. From 15 to 30 μg of total protein, cytoplasmic protein, or nuclear protein was resolved by SDS/PAGE and then transferred onto a polyvinylidene difluoride (PVDF) membrane. The membrane was washed with Tris-buffered saline Tween (TBS-T, 20 mM Tris-HCl, 150 mM NaCl, and 0.05% Tween 20). Non-specific sites on the membrane were blocked by incubating the membrane in blocking solution containing 5% non-fat dry milk in TBS-T for 60 min at room temperature. The membrane was then washed and incubated with diluted respective primary antibodies (anti-iNOS, anti-COX-2, anti-IκB-α, anti-p65, anti-p-ERK, anti-p-JNK and anti-p-p38 antibodies) at 4 °C overnight. The membrane was washed and incubated in a solution containing HRP-conjugated secondary antibody for 1 h. After the final washing, the membrane was reacted with enhanced chemiluminescence (ECL) reagent to allow the detection of proteins with a Luminescent Image Analyzer (LAS-3000, Fujifilm, Tokyo, Japan). Densitometric values were normalized using β-actin.

### 4.4. In Vivo Study

#### 4.4.1. Animals

Adult male Balb/c mice (22–25 g) were purchased from Orient Bio Co. (Sungnam, Korea). All mice were fed a standard laboratory diet and given water *ad libitum*, and were housed under standard laboratory conditions of controlled lighting (12 h light, 12 h dark) and temperature (21 ± 1 °C). All animal care procedures and experiments were approved by the Institutional Animal Care and Use Committee of Konkuk University, in accordance with the principles and guidelines of the U.S. National Institute of Health Guide for the Care and Use of Laboratory Animals.

#### 4.4.2. Murine Model and Grouping of LPS-Induced ALI

Mice were randomly divided into five groups, as detailed below. Each group contained eight mice.

Group INegative controlGroup IILPS control (5 mg/kg)Group IIILPS + CF (25 mg/kg)Group IVLPS + CF (50 mg/kg)Group VPositive control (LPS + Dexamethasone 10 mg/kg)

CF and dexamethasone were administered intraperitoneally twice at the interval of 12 h. Two hours later after the last the CF and dexamethasone injection, mice were slightly anesthetized with an injection of avertin and LPS (5 mg/kg) in 40 μL PBS was instilled intranasally (i.n.) to induce lung injury. Control mice were given 40 μL PBS without LPS. All the mice were alive 24 h after the LPS treatment.

#### 4.4.3. Bronchoalveolar Lavage Fluid (BALF) Collection

Mice were sacrificed at selected times after CF administration and LPS challenge. BALF was obtained by intratracheal instillation, and the lungs were lavaged twice with 1.0 mL sterile saline each time through the tracheal cannula. The collected BALF was placed directly on ice and then centrifuged at 2000 rpm for 3 min at 4 °C. The cell-free supernatant was used to measure total protein content and cytokine concentration assay. The sedimented cells were resuspended in PBS. Total cell count was obtained using a hemocytometer. The percentage of neutrophils was determined by Gr-1 staining using flow cytometry, after which the numbers of neutrophils were calculated by total cell count × percentage of neutrophils.

#### 4.4.4. Histopathological Analysis

Evaluation of lung injury, including alveolar congestion, hemorrhage, infiltration of leukocytes, change in thickness of the alveolar wall, and formation of a hyaline membrane, was performed using histological analysis [28]. Dissected lungs were washed, fixed with 4% paraformaldehyde, and embedded in paraffin. The paraffin-embedded lung tissues were then sliced into 5 μm sections, which were used in histological analysis. Lung tissue sections were stained with hematoxylin and eosin to delineate the inflammatory response and pathological changes in the lung tissue. The lung injury score was categorized according to the sum of the damage level such as thickening of alveolar walls and epithelium, the numbers of infiltration cells, as well as increases in peribronchial and perivascular cuff area. Each histological characteristic was scored 0 to 5.

#### 4.4.5. Measurement of Cytokines

The levels of TNF-α, and IL-6 in the BALF collected from mice were measured using ELISA kits (R & D Systems), according to the manufacturer’s instructions.

### 4.5. Statistical Analysis

Data were reported as means ± standard deviation from triplicate determinations. Analysis of variance (ANOVA), accompanied with Dunnett’s tests (GraphPad Prism 5), was conducted to identify the significant differences between samples (*p* < 0.05).

## 5. Conclusions

Evaluation of the anti-inflammatory effects of CF using LPS-stimulated RAW264.7 macrophages revealed that CF markedly suppressed the generation of IL-6 and TNF-α, and inhibited the protein expression of iNOS and COX-2, as well as the consequent secretion of NO, with a strong reduction in the intracellular ROS level. The results suggest that CF can modulate inflammatory processes through the NF-κB pathway and MAPKs activation. *In vivo*, CF suppressed LPS-induced over-production of neutrophils and inhibited the production of cytokines such as IL-6 and TNF-α in the BALF. Overall, our findings indicate that CF may have potential for use in the treatment of inflammation-associated diseases.
